# Current Views about the Inflammatory Damage Triggered by Bacterial Superantigens and Experimental Attempts to Neutralize Superantigen-Mediated Toxic Effects with Natural and Biological Products

**DOI:** 10.3390/pathophysiology31010002

**Published:** 2024-01-09

**Authors:** Luigi Santacroce, Skender Topi, Ioannis Alexandros Charitos, Roberto Lovero, Paolo Luperto, Raffaele Palmirotta, Emilio Jirillo

**Affiliations:** 1 Section of Microbiology and Virology, Interdisciplinary Department of Medicine, School of Medicine, University of Bari ‘Aldo Moro’, 70124 Bari, Italy; emilio.jirillo@uniba.it; 2 Department of Clinical Disciplines, University ‘Alexander Xhuvani’ of Elbasan, 3001 Elbasan, Albania; 3 Division of Pneumology and Respiratory Rehabilitation, Maugeri Clinical Scientific Research Institutes (IRCCS) of Pavia—Scientific Institute of Bari, 70124 Bari, Italy; 4 Clinical Pathology Unit, AOU Policlinico Consorziale di Bari-Ospedale Giovanni XXIII, 70124 Bari, Italy; 5 Brindisi Local Health Agency, 72100 Brindisi, Italy

**Keywords:** superantigens, toxic shock syndrome (TSS), immune response, antimicrobial peptides, beta-glucans, cytokines, autoimmunity, polyphenols, probiotics

## Abstract

Superantigens, i.e., staphylococcal enterotoxins and toxic shock syndrome toxin-1, interact with T cells in a different manner in comparison to conventional antigens. In fact, they activate a larger contingent of T lymphocytes, binding outside the peptide-binding groove of the major histocompatibility complex class II. Involvement of many T cells by superantigens leads to a massive release of pro-inflammatory cytokines, such as interleukin (IL)-1, IL-2, IL-6, tumor necrosis factor-alpha and interferon-gamma. Such a storm of mediators has been shown to account for tissue damage, multiorgan failure and shock. Besides conventional drugs and biotherapeutics, experiments with natural and biological products have been undertaken to attenuate the toxic effects exerted by superantigens. In this review, emphasis will be placed on polyphenols, probiotics, beta-glucans and antimicrobial peptides. In fact, these substances share a common functional denominator, since they skew the immune response toward an anti-inflammatory profile, thus mitigating the cytokine wave evoked by superantigens. However, clinical applications of these products are still scarce, and more trials are needed to validate their usefulness in humans.

## 1. Introduction

Daily, a myriad of noxious antigens enters the host through both respiratory and intestinal tracts. However, the mucosal immune arsenal plays a protective role against pathogenic microorganisms and their excreted products [1,2,3]. Bacteria and their toxins, which can cause certain diseases (some already known in ancient medicine such as that caused by *Corynebacterium diphtheriae*), are the most frequent invaders of humans and animals, but the host can trigger a sequence of defensive immunological events. A first defense line is represented by the innate immune cells, while for more specific responses a second category of cells intervene, i.e., T and B lymphocytes, which govern the so-called adaptive immunity. In this framework, pathogen-associated molecular patterns (PAMPs) expressed on the surface of both Gram-positive and Gram-negative bacteria bind specific receptors on the surface of monocytes–macrophages and neutrophils, thus eliciting a pro-host response [4,5]. Quite importantly, innate immune cells do not retain immunological memory, which is a prerogative of the adaptive immunity, composed of various subsets of T and B cells. Nevertheless, as recently reported [6], phagocytes, as major components of the innate immunity, are endowed with so-called trained immunity, which allows a faster response towards a reencounter with the same pathogen. On the other hand, T cells respond to antigens presented in the context of the MHC class II molecules, with T cytotoxic (CD8+) cells, as killers of infected cells and T helper (h) (CD4+) cells, as supporters of memory CD8+ cell responses [7,8,9]. Furthermore, Th cells can kill pathogens activating macrophage-mediated phagocytosis and B cell-induced production of specific antibodies [10]. T cells are endowed with alpha-beta T cell receptors (TCRs) to detect foreign antigens presented by antigen-presenting cells (APCs), e.g., macrophages and dendritic cells (DCs), through the major histocompatibility complex (MHC), class I or class II [11]. Then, following antigen presentation, naïve alpha-beta T cells become activated effector T cells with the participation of cytokines and costimulatory signals [12,13]. In the case of microorganisms, T cell response leads to the elimination of a given pathogen with the formation of memory cells, which harbor in the blood, lymphoid organs and mucosal sites and are maintained by interleukin (IL)-7 and IL-15 [14,15,16]. Therefore, re-exposure to the same pathogen leads to a prompter and more effective response. Conversely, the so-called superantigens, i.e., staphylococcal enterotoxins (SEs) and toxic shock syndrome toxin-1 (TSST-1), interact with T cells in a different manner. The superantigen family includes several 22–29 kDa proteins, highly resistant to proteases and heat denaturation. Analysis of the three-dimensional structure of superantigens reveals a common molecular architecture, consisting of a C-terminal domain with a ubiquitin-like motif and an N-terminal domain with a characteristic *OB* fold that binds oligosaccharides and oligonucleotides (oligosaccharide/oligonucleotide-binding fold), separated by a long α-helix extending down the center of the molecule. They are unconventional antigens, which trigger a response by binding outside the complementary determining regions of their target immune receptor macromolecules (antibodies or T cell receptors [17]. In fact, they activate a large contingent (5–30%) of T cells in comparison to conventional antigens, which stimulate <0.01% of T cells [18]. Furthermore, superantigens bind outside the peptide-binding groove of MHC class II without internalization and processing and are not MHC class II-restricted. The binding of the superantigen/MHC II complex to specific V-beta regions of TCR represents the first signal for T cell activation that is followed by binding of costimulatory molecules, as a second signal, with an early cytokine storm and massive polyclonal T cell proliferation [19]. In fact, superantigens stimulate human peripheral blood mononuclear cells (PBMCs) with early release of a plethora of cytokines, such as IL-1, Il-2, Il-6, tumor necrosis factor (TNF)-alpha and interferon (IFN)-gamma [20]. In turn, IL-1 and TNF-alpha activate other cells, as well as tissue factor and matrix-metalloproteinases, thus contributing to damage of the immune and cardiovascular system, culminating in multiorgan dysfunction and shock [21,22].

Over the recent decades, besides the use of conventional drugs and monoclonal antibodies to limit the superantigen-mediated injury, experimental studies with natural and biological products have been carried out. However, clinical attempts to evaluate their putative antimicrobial and anti-inflammatory role against superantigen effects are still scarce, but to date some promising experimental results have been published. In this framework, polyphenols are compounds largely present in the vegetal kingdom that are currently used in the treatment of several chronic inflammatory diseases [23]. Probiotics and, mostly, lactobacilli, when administered, provide the host with beneficial effects in terms of anti-inflammatory and antimicrobial activities [24]. Beta-glucans present in mushrooms and bacteria inhibit the activation of NF-kB with diminished release of pro-inflammatory cytokines [25].

Among biological products, antimicrobial peptides (AMPs) are produced by epithelial cells and innate immune cells, and they are very effective against a broad range of bacteria, even including antibiotic resistant bugs [26,27].

In the light of the above notions, the present review is aimed at describing the mechanisms of interactions of superantigens with host cells and related inflammatory damage. Then, the use of polyphenols, beta-glucans, probiotics and AMPs in different experimental settings to block the toxic activity of superantigens will be described.

## 2. T Cell Receptor and T Cell Activation

According to the TCRs expressed on their membrane, T cells can be divided into alpha-beta or gamma-delta T cells that play different functions [28,29,30]. Alpha-beta T cells recognize small peptide antigens on the cell surface of antigen-presenting cells (APCs) complexed to MHC class I or class II molecules. Usually, CD4+ T cells recognize MHC class II molecules, while CD8+ T cells recognize MHC class I molecules, even if the latter are also able to recognize MHC class II molecules [28,31]. On the other hand, gamma-delta T cells recognize antigens in an MHC-unrestricted manner, engaging CD1 molecules that are encoded outside the MHC locus and able to present microbial lipids to T cells [32].

With special reference to alpha-beta T cells, they can respond to an enormous array of microbial antigens in view of a diversity of TCR sequences that are based on alpha- and beta chain rearrangements [33]. Then, the so-called clonotypic diversity relies on somatic recombination of V, D and J gene segments and junctional adaptations, which create three complementary determining region (CDR) loops [34]. CDR1 and CDR2 loops bind the TCR to its target MHC, whereas hypervariable loops engage the exposed regions of the MHC bound peptide. The above mechanisms of clonal expansion can enhance the numbers of antigen-specific effector T cells [35].

Briefly, in response to microbial antigens, naïve alpha-beta T cells differentiate into Th1, Th2, Th17, T regulatory (TREG) cellular subsets, with Th1 lymphocytes governing the cellular immunity and Th2 cells directing the humoral immunity [36]. CD4+ Th1 cells use T-bet, STAT1 and STAT4 for transcriptional regulation, with release of IL-2, IFN-gamma, TNF-alpha and IL-12, thus leading to a protective response against intracellular pathogens. IL-2 and IFN-gamma help CD8+ cells and phagocytes in bacterial killing [37]. On the other hand, CD4 + Th2 cells utilize GATA3, STAT5 and STAT6 for transcriptional regulation and produce IL-4, IL-5 and IL-13 for antibody production against extracellular pathogens, even including parasites. Memory CD8+ T cells generate fast recall responses to bacterial antigens while maintaining long-term immunity [38]. Memory cells have been divided into central memory T, effector memory T and tissue resident memory T (TRM) cells [39]. CD8+ TRM cells mostly protect against pathogens that invade mucosal tissues via local proliferation, with release of IFN-gamma, TNF-alpha and cytotoxic molecules, such as granzyme B [40]. Gamma-delta T cells express TCRs composed of rearranged TCR-gamma and TCR-delta chains distinct from alpha-beta TCRs. They are encoded by a lower number of V, D and J segments with a limited clonotypic diversity [41]. Functionally, gamma-delta T cells rapidly respond to microbial antigens, with the production of IFN-gamma, TNF-alpha and IL-17 [42]. It has been reported that gamma-delta T cells from long-lived memory cells, following bacterial challenge, can permanently reside in the affected tissue.

## 3. Modalities of Superantigen Binding to TCR and Induction of the Inflammatory Pathway

Superantigens bind outside the peptide-binding groove on MHC molecules through two distinct binding sites [43,44,45]. In this respect, the invariant alpha chain of MHC II is used by the majority of superantigens, while, among others, SEA and SED bind the polymorphic beta chain [46,47,48]. The binding of the superantigen/MHC II complex to TCR represents the first signal for T cell activation to occur, while the second signal is delivered by the binding of costimulatory molecules CD80 and CD86 on APCs with CD28 expressed on T cells [49,50,51,52,53]. Furthermore, the intervention of CD2, intercellular adhesion molecule-1 (ICAM-1) and endothelial adhesion molecule activate endothelial cells and T cells via SEB, while CD11a/ICAM-1 and CD28/CD80 stimulation activate T cells via SEA [51,54]. All the above-described events culminate in the activation of protein tyrosine kinases (PTKs) and of calcineurin phosphatase with translocation of the nuclear factor of activated T cells to the nucleus that, in turn, enhances the expression of IL-2 and other T cell-derived cytokines [54]. Among the transcriptional factors activated, NF-kB binds the promoter region of several pro-inflammatory cytokines, T cell growth and differentiation factors, thus leading to the release of a variety of mediators outside cells, as an expression of the third signal [55]. In this scenario, the role played by NOD-like-receptors (NLRs), and particularly NLRP3, as triggers of pro-inflammatory cytokines needs to be clarified. Of note, the NLR inflammasome is an essential component of the innate immune system, leading to caspase-1 activation, with secretion of IL-1 beta and IL-18 in response to microbes and or their toxins [55]. Recent in vitro study has demonstrated that recombinant TSST-1 could activate NLRP3 inflammasome in murine primary macrophages [56]. Release of IL-1 beta and TNF-alpha by stimulated macrophages occurred in the presence of Toll-like receptor (TLR)-4, as a first signal.

Cytokines represent the main players of superantigen-induced damage. Among them, IL-1 and TNF-alpha contribute to extending the inflammatory status, increasing vascular permeability and, ultimately, provoking multiorgan failure and shock [57]. Furthermore, the chemokines, IL-8, monocyte chemoattractant protein-1, macrophage inflammatory protein (MIP)-1 alpha and MIP-1 beta, are induced via SEA, SEB and TSST-1 [58,59]. Systemic and intranasal exposure to SEB has been shown to induce infiltration of neutrophils and mononuclear cells into tissue with increased vascular permeability and acute lung injury (ALI) [60,61]. Furthermore, superantigens can also drive polyclonal IgE production, the so-called “T-cell dependent super allergen” with degranulation of mast cells and basophils [62,63]. For instance, SE sensitization was associated with allergic poly-sensitization against food and inhaled allergens in adolescents [64].

**Figure 1:**
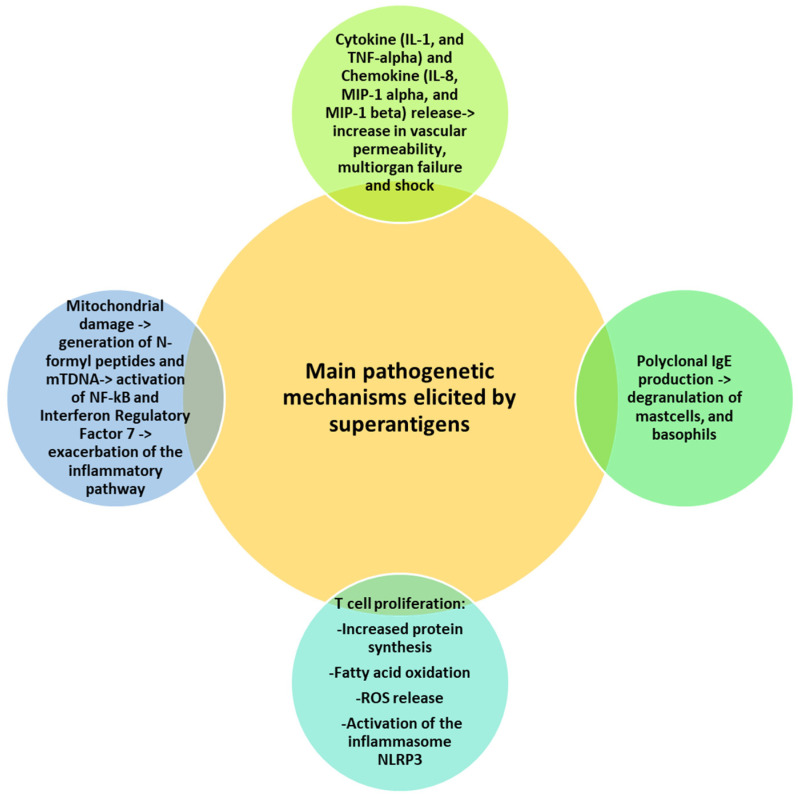
Superantigen-mediated damage. Various mechanisms cooperate in the superantigen-induced harmful effects, including release of cytokines and chemokines, T cell proliferation and mitochondrial damage. All mechanisms share a common denominator represented by the induction of the inflammatory pathway.

Massive T cell proliferation induced by superantigens is followed by metabolic activities, such as increased protein synthesis, fatty acid oxidation and reactive oxygen species (ROS) generation with endoplasmic reticular (ER) stress and mitochondrial damage [65]. ER stress activates the inflammasome NLRP3, with further release of IL-1 beta in a caspase 8-dependent manner [66]. Superantigen-induced mitochondrial damage promotes the release of cytochrome c, ATP, N-formyl peptides (NFPs) and mtDNA [67]. NFPs are chemoattractants for neutrophils, while mtDNA binds endosomal TLR9 and activates NF-kB and interferon regulatory factor 7, thus exacerbating the inflammatory pathway [68]. Figure 1 summarizes the main mechanisms of superantigen-mediated damage.

## 4. Superantigen-Mediated Disease

The most frequent disease caused by superantigens is toxicosis, especially that associated with staphylococcal food poisoning with nausea and vomiting [69,70]. It seems that superantigens bind a yet unidentified receptor in the gut, thus evoking release of 5-hydroxy tryptamine that, in turn, leads to the vomiting reflex through depolarization of the vagal afferent nerves.

Penetration of superantigens into the bloodstream causes TSS, characterized by high fever, inflammation, vascular leakage, hypotension, erythematous rash and multiorgan failure [57]. Notably, TSS is a rare disease, since humans possess high titers of neutralizing antibodies [71].

Kawasaki syndrome (KS) is an acute multisystem vasculitis in children with coronary artery lesions [72,73]. Even if the etiology of KS is still unknown, many similarities have been observed between KS and TSS [74]. The common pathogenetic denominator seems to rely on the cytokine storm they elicit with hypotension, vascular capillary leak syndrome and vasculitis. Quite interestingly, COVID-19 infection in children seems to share clinical symptoms with KS and TSS and occurs 4–5 weeks post-COVID-19 [75].

Furthermore, it is noteworthy that some superantigens, such as staphylococcal or mycoplasmal toxins, can induce nonspecific activation of large subsets of T cells directly binding to MHC molecules expressed on them, without processing. Because the peptides expressed on the cell together with the MHC proteins derive not only from antigens but also from cellular metabolism, being “self-molecules” of the organism itself can trigger an autoimmune response which is expressed with different clinical pictures (e.g., systemic lupus erythematosus, rheumatoid arthritis and inflammatory bowel disease) [73].

## 5. Natural and Biological Products as an Alternative Treatment of Superantigen-Mediated Damage

Treatment of superantigen-induced toxic shock is still limited. Experimental attempts have been undertaken with immunosuppressants, such as dexamethasone, cyclosporine A and rapamycin with the aim of interrupting T cell proliferation [53,76,77,78,79]. Furthermore, intravenous immunoglobulin and, recently, humanized monoclonal antibodies (MoAbs) to neutralize SEs and TSST-1 have been used [80,81,82,83]. However, MoAbs exert a limited inhibition of the binding of superantigens to their receptors in the early phase of toxin exposure and, therefore, a downstream blockade seems to be the only means of intervening, in order to inhibit the cytokine storm. Of note, most of the studies are still experimental, and mice represent the best model to investigate the immunological involvement during superantigen-mediated shock [84]. In parallel, the potential ability of natural and biological products to attenuate the superantigen-induced damage in in vitro and in in vivo experimental models is under investigation, as described below. 

### 5.1. Polyphenols

Polyphenols are largely present in the vegetal kingdom, especially in fruits, vegetables, cereals, extra virgin olive oil and red wine. There is a large body of evidence that polyphenols exert antioxidant and anti-inflammatory activities, and, for this reason, they are currently used for the treatment of various chronic disease [85,86,87]. Among major mechanisms of action, polyphenols can inhibit the activation of the NF-kB pathway with dampening of proinflammatory cytokine release while activating T regulatory (TREG) cells with production of the anti-inflammatory cytokine IL-10 [88,89]. A few experimental data are available about the effects of polyphenols on superantigens.

Resveratrol (RES) is a stilbene that has been investigated during superantigen-mediated acute respiratory syndrome (ARDS) [90]. SEB administration induced release of proinflammatory cytokines and ARDS in mice, increasing Proteobacteria phylum and *Cutibacterium acnes* (formerly *Propionibacterium acnes)* species in the lung. RES treatment mitigated the inflammatory profile and reduced mortality, also increasing beneficial bacteria in the colon and lungs, such as *Limosilactobacillus reuteri* (previously named *Lactobacillus reuteri).* In another study, severity of SEB-mediated ALI in mice was mitigated by RES treatment through regulation of miR-193a and induction of the anti-inflammatory cytokine, transforming growth factor (TGF)-beta [91]. Altogether, these data demonstrate the ability of RES to reduce the inflammatory process during ARDS and ALI. Involvement of TGF-beta via RES represents a potential mechanism of immunosuppression, even including its ability to induce TREG cell activation [92]. In another study, apple juice and apple polyphenols inhibited the biological activity of SEA, without any cytotoxic effects on spleen cells [93]. SEA was irreversibly bound to apple juice constituents, thus suggesting the possibility to use polyphenols in vivo. Furthermore, it has been reported that epigallocatechin gallate (EGCG) contained in green tea could block peripheral blood mononuclear cell activation via SEB, preventing the dysfunction of the intestinal epithelial barrier [94].

### 5.2. Beta-Glucans

B-1-3-D-glucans (beta-glucans) represent main components of the microbial cell wall or can be secreted by fungi, such as *Saccharomyces cerevisiae* or *Candida albicans* [95]. Beta-glucans bind a specific receptor, dectin-1, on dendritic cells, monocytes–macrophages and neutrophils, whose binding accounts for activation of the NF-kB pathway with production of pro-inflammatory cytokines [96,97,98]. Conversely, in a murine polymicrobial sepsis model, beta-glucan treatment diminished morbidity and mortality, inhibiting NF-kB, while triggering phosphoinositide-3-kinase. With special reference to superantigens, PBMCs were exposed to glucan phosphate (GP) and then stimulated with TSST-1 for 48 h [99]. Determination of cytokine production demonstrated that GP could lead to an anti-inflammatory shift with production of IL-1 receptor antagonist. Such a protective function exerted by GP in vitro was supported by in vivo data of beta-glucan-mediated cardio protection [100]. In another study, stimulation of murine lymphocytes isolated from soluble beta-glucan-treated mice with SEB or TSST-1 increased production of IFN-gamma while abrogating production of IL-2 and TNF-alpha [101]. These results indicate that beta-glucans may represent immunomodulators for controlling release of pro-inflammatory cytokines from superantigen-activated lymphocytes and monocytes with mitigation of septic shock.

### 5.3. Lactobacilli

Lactobacilli are commensal bacteria belonging to the class of probiotics. They are dietary supplements that provide the host with beneficial effects, acting as immunomodulators [102,103]. In this respect, Lactobacilli, as well as their cell-free supernatants, exert different functions, such as induction of TREG cells with production of IL-10 and a shift towards an anti-inflammatory profile [104,105]. A few studies have been focused on the ability of lactobacilli to neutralize the toxic effects of SE. Colonization of *S. aureus* in early life was associated with a massive release of cytokines, while co-colonization with lactobacilli in vitro decreased immune response [106]. Also, soluble factors derived from lactobacilli were able to reduce the *S. aureus*-induced activation of T cells and release of pro-inflammatory cytokines [107]. In another study, PBMCs were cultured with *S. aureus* cell-free supernatants (CFSs) and SEA in the presence of *L. rhamnosus* CFS and *L. reuteri* [108]. Then, activation of T cells and natural killer cells was evaluated. Results showed that these cells were activated by *S. aureus*-CFS, while SEA and CFS derived from lactobacilli in vitro decreased immune functions through direct cellular contact. In fact, immune suppression occurred in the absence of antigen presentation or APC-derived IL-10. Furthermore, during *S. aureus* bloodstream infection in mice, an exopolysaccharide (EPS) from *Bacillus subtilis* reduced mortality [109]. In this test system, EPS abrogated the IL-12-derived production of IFN-gamma by NK cells in a TLR-4-dependent manner. 

**Figure 2:**
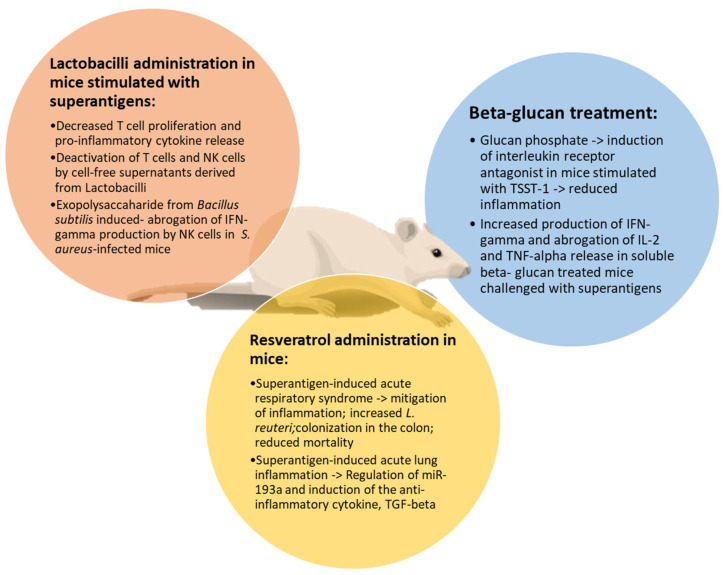
Main effects of natural products on superantigen-induced damage. Resveratrol, beta-glucans and lactobacilli share common activities in attenuating inflammation, reducing T cell proliferation and cytokine release in mice stimulated by superantigens.

In Figure 2, the effects of natural products against superantigen-mediated damage are illustrated.

### 5.4. Antimicrobial Peptides

AMPs are small peptides produced by innate immune cells that exert broad antibacterial activity against both Gram-positive and Gram-negative bacteria [26,27]. The emergence of bacteria resistant to antibiotics represents a serious health care problem, and AMPs are currently used as a potential alternative treatment to antibiotics [110]. Very few studies focused on the ability of AMPs to neutralize superantigens.

Evidence has been provided that naturally occurring hemoglobin alpha chain peptides can inhibit TSST-1 from *S. aureus*, likely interfering with plasma membrane signal transduction in view of their positive charges [111]. Moreover, this AMP was nontoxic to human vaginal cells, without inhibiting the normal microbiotal member *L. crispatus* that produces anti-inflammatory mediators. In another report, expression of IL-26 has been demonstrated in skin wounds infected with *S. aureus* [112]. In this context, SE was responsible for IL-26 expression in T cell lines and primary skin T cells. Il-26 was able to inhibit growth of *S. aureus* and biofilm formation.

In conclusion, IL-26 behaves as an AMP, hampering T cell responses to SE. Lactoferrin (LF) is an AMP that is largely present in colostrum, milk and other exocrine secretions [113,114]. LF has bacteriostatic and bactericidal activities in view of its ability to chelate iron and prevent LPS binding to TLR-4 [115]. Bovine (b)LF could mitigate SEB- induced proliferation, IL-2 release and CD25 expression by transgenic murine T cells [116]. Furthermore, bLF hampered cytokine secretion from Jurkat T cell lines and PBMCs from healthy donors in response to SEB. In the light of these findings, bLF may represent a potential therapeutic tool to protect the host during the toxic shock syndrome. Conversely, it has been reported that human transferrin and LF could modulate expression of streptococcal pyrogenic exotoxins (Spes) [117]. Down-regulation of SpeB may favor bacterial growth, preserving the integrity of M proteins and superantigens [118,119].

**Figure 3:**
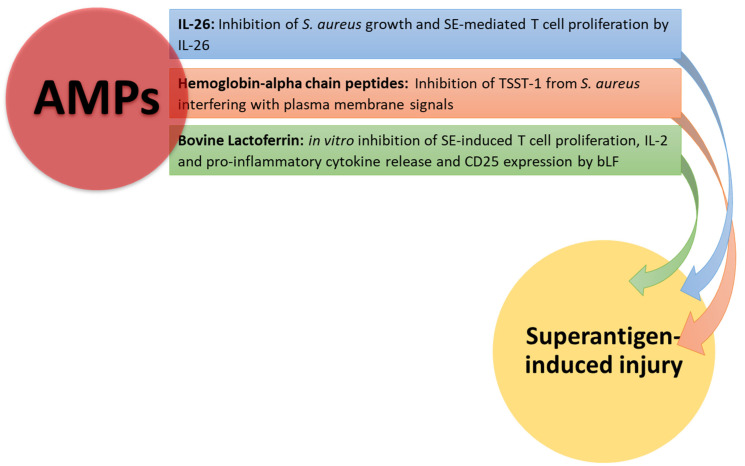
AMP neutralizing effects on superantigen-mediated damage. Hemoglobin-alpha chain peptides and IL-26 inhibit *S. aureus* growth, interfering with plasma membrane signals. Il-26 and bLF inhibit T cell proliferation and cytokine production by T cells stimulated with SEB.

In Figure 3, the effects of AMPs on superantigen-induced injury are shown.

## 6. Conclusions

Superantigens are very dangerous bacterial products, since they bind the TCR on T cells outside the peptide-binding groove, thus involving a far larger number of lymphocytes. Binding is followed by the liberation of the cascade of pro-inflammatory cytokines, which may lead to a condition of multiorgan failure and death. Attempts to block the above cited binding and neutralize the liberation of pro-inflammatory cytokines systemically with the use of immunosuppressants and biotherapeutics, such as MoAbs, are under investigation. However, clinical trials are still scarce, and drugs like MoAbs can induce side effects. On these grounds, the attention of scientists has recently been focused on the use of natural and biological products as potential anti-inflammatory and antimicrobial agents. In fact, polyphenols, beta-glucans, probiotics and AMPs share common activities in terms of inhibition of the NF-kB pathway and release of anti-inflammatory cytokines, mainly IL-10, also arresting the growth of pathogenic bacteria. Moreover, they exert immunomodulating activities, mitigating the noxious effects of superantigens. The advantages of these products are the absence of side effects and limited costs in comparison to MoAbs. Despite that, there are very few clinical trials, and, therefore, exploitation of the properties of natural and biological products against superantigen-mediated damage is needed. Natural products may represent a prospective therapeutic tool to prevent or mitigate the deleterious effects exerted by superantigens. 

## Data Availability

All available data have been reported in the manuscript.

## Abbreviations

ALI	Acute lung injury
AMPs	Antimicrobial peptides
APCs	Antigen-presenting cells
b-LF	Bovine lactoferrin
CDR	Complementary determining regions
CFs	Cell-free supernatants
DCs	Dendritic cells
EPs	Exopolysaccharides
ER	Endoplasmic reticular
ICAM-1	Intercellular adhesion molecule-1
IFN	Interferon
IL	Interleukin
LF	Lactoferrin
MHC	Major histocompatibility complex
MIP-1	Macrophage inflammatory protein-1
NFPs	N-formyl peptides
NLRs	Nod-like receptors
PAMPS	Pathogen-associated molecular patterns
PTKs	Protein tyrosine kinases
RES	Resveratrol
ROS	Reactive oxygen species
SEs	Staphylococcal enterotoxins
SPEs	Streptococcal pyrogenic exotoxins
Th	T helper
TGF-beta	Transforming growth factor-beta
TLRs	Toll-like receptors
TNF-alpha	Tumor necrosis factor-alpha
TREG	T regulatory
TRM	T resident memory
TSS	Toxic shock syndrome
TSST-1	Toxic shock syndrome toxin-1
